# Assessment of retinal vascular oxygenation and morphology at stages of diabetic retinopathy in African Americans

**DOI:** 10.1186/s12886-020-01566-y

**Published:** 2020-07-18

**Authors:** Sarah L. Garvey, Maziyar M. Khansari, Xuejuan Jiang, Rohit Varma, Mahnaz Shahidi

**Affiliations:** 1 College of Medicine, University of Illinois at Chicago, Chicago, IL USA; 2 Department of Ophthalmology, University of Southern California, 1450 San Pablo Street, Los Angeles, California 90033 USA; 3 Stevens Neuroimaging and Informatics Institute, University of Southern California, Los Angeles, CA USA; 4 Southern California Eye Institute, CHA Hollywood Presbyterian Medical Center, Los Angeles, CA USA

**Keywords:** Diabetic retinopathy, African American, Oxygenation, Tortuosity, Image analysis

## Abstract

**Background:**

Diabetic retinopathy (DR) is a microvascular complication of diabetes and a leading cause of blindness in working-age adults. The likelihood of visual impairment associated with DR is two-fold higher in the African-American (AA) compared to non-Hispanic white. Although alterations in retinal vessel oxygenation and morphology have been reported in DR, there is limited knowledge about these vascular changes in AA subjects. The purpose of the current study was to investigate alterations in retinal vascular oxygen saturation (SO_2_), vessel diameter (D) and tortuosity at severity stages of DR in AA subjects.

**Methods:**

A nested case-control study of 56 AA subjects was conducted. Right eyes were grouped as non-diabetic (ND) (*N* = 26), no clinical DR (NDR) (*N* = 19), or moderate/severe non-proliferative DR (NPDR) (*N* = 11). Imaging was performed using a commercially available scanning laser ophthalmoscope. Images were analyzed to determine retinal arterial and venous SO_2_ (SO_2A_ and SO_2V_), diameter (D_A_ and D_V_), and vessel tortuosity index (VTI) (VTI_A_ and VTI_V_).

**Results:**

SO_2V_ and D_V_ were higher in NPDR compared to ND and NDR groups (*P* < 0.05). There were no significant differences in SO_2A_ and D_A_ among ND, NDR, and NPDR groups (*P* > 0.8). Maximum VTI_A_ was higher in diabetics (NDR and NPDR) compared to non-diabetics (*P* < 0.03). There was no significant difference in maximum VTI_V_ among the 3 groups (*P* = 0.5).

**Conclusions:**

The findings advance our understanding of DR pathophysiology in the AA population and may propel identification of race-specific retinal vascular biomarkers for improved diagnosis and monitoring of DR.

## Background

Diabetic retinopathy (DR), a microvascular complication of diabetes, is one of the leading causes of blindness in the United States [1]. DR subjects are 29 times more likely to go blind as compared with non-diabetics of similar age and gender [2]. While DR has been associated with increased duration of diabetes and HbA1c levels [3], limited knowledge is available regarding the development of retinal pathologies. The established risk factors partially explain the pathogenesis of DR [4], therefore identifying additional quantifiable markers becomes important in better understanding the development, diagnosis and management of DR. According to the Model Reporting Area (MRA) blindness registry data, a comparison of age-adjusted rates of legal blindness associated with DR demonstrated a two-fold increased likelihood of blindness for predominantly African-American (AA) as compared to non-Hispanic white (NHW) [5]. There has also been a rise in the incidence of diabetes in AA population [6, 7]. Type 2 diabetics constitute 95% of AA with diabetes, and the incidence of type 2 diabetes in AA is 50 to100% greater than in NHW [8]. Despite the fact that racial minorities in the United States have experienced an increased rate of diabetes, race-specific data on the development of blindness and DR in AA populations is scarce.

In addition to increased prevalence of diabetes, AA experience higher rates of morbidity and mortality relative to NHW with diabetes [9]. This includes an increased risk of diabetes-related microvascular complications such as end-stage nephropathy, diabetic retinopathy, and peripheral neuropathy. The increased incidence of diabetes-related limb amputations and end-stage nephropathy in the AA population is well-documented [10]. However, there is comparably little data regarding the racial differences in the rate of DR. Most of the existing studies on the population-based epidemiology and risk factors for developing DR have involved insufficient number of AA, and therefore have been unable to provide race-specific details regarding the development of this disease in this population [11]. Nevertheless, the Salisbury Eye Evaluation Study [12] provided comparative race-specific data for visual impairment due to DR in AA and NHW. They found that AA were 4 times as likely to develop DR as compared with NHW, and that DR accounted for 17% of vision impairment as compared with 8% in NHW. They concluded that DR is the second leading cause of vision impairment in the AA population. These results reflect the substantial impact of DR and vision loss in AA and necessity of further investigation.

One quantifiable marker of DR is based on assessment of the retinal vasculature. Recent studies have shown that changes in retinal vessel caliber and dimension are linked to diabetes [13–15]. Furthermore, changes in retinal hemodynamics such as decreased blood flow, disruption of vascular endothelium, and increased vascular endothelial growth factor (VEGF) affect retinal vessel tortuosity [16, 17]. However, reported changes in retinal blood flow in DR have been variable [18–20]. Increased retinal vessel tortuosity has been reported in diabetes [21, 22] and increased retinal arteriolar tortuosity was found to be associated with a high HbA1c levels in patients with type 1 diabetes, even in young patients with no DR. [23] However, limited quantitative data is available regarding tortuosity of retinal vessels in AA at stage of DR.

Another vital component of the pathophysiology of DR is abnormal retinal oxygenation [24]. This was determined by the treatment of neovascularization with VEGF inhibitors and vascular non-perfusion on fluorescein angiography in hypoxia-induced states [25, 26]. Oxygenation of the retina depends on the rate of blood flow and oxygen concentration within blood. Previous studies have shown that blood flow velocity is decreased in retinal arteries and veins in early diabetes [27]. Recent studies have reported increase in vessel diameter in diabetic subjects [28–30]. Meanwhile the concentrations of oxygen in the arterial and venous blood are directly related to the oxygen saturations of hemoglobin (SO_2_). SO_2_ can be measured by comparing absorption of light by oxy- and deoxy-hemoglobin at 2 different wavelengths. Several studies have demonstrated alterations in retinal vascular SO_2_ due to diabetes [31]. However, these studies featured a population that was mostly NHW and homogeneous, though one recent study reported an effect of race on SO_2_ measured in DR subjects [24]. The purpose of the current study was to investigate alterations in retinal vascular SO_2_, diameter and tortuosity at stages of DR in an AA cohort from the African American Eye Disease Study (AFEDS).

## Methods

### Subjects

The study was approved by the Institutional Review Board of the University of Southern California and was conducted in accordance to the Tenets of Declaration of Helsinki. The study included 56 AA subjects (female/male = 60%/40%) who participated in the AFEDS, with ages ranging from 40 to 87 years old. By July 2017, a total of 14 subjects with moderate/severe NPDR and 18 diabetic subjects without clinical DR (NDR) were identified to have images acquired by Optos Daytona in the AFEDS study. Images were reviewed by an ophthalmologist and those with poor quality due to media opacities or artifacts (e.g. eye lash, dust spots) were excluded. All diabetic subjects were considered to have type II diabetes based on not being insulin dependent or had been diagnosed with diabetes after 30 years of age. The duration of diabetes was calculated as the difference between the year of diagnosis (self-reported) and the year of vision examination in AFEDS. An equal number of subjects (*N* = 32) without diabetes (ND) were randomly selected from the AFEDS study. Data on vascular oxygen saturation and vessel dimeter were available in 26 ND, 19 NDR, and 11 NPDR. Only 2 NPDR subjects had macular edema. DR severity was determined as part of the AFEDS study in a masked manner at the Grading Center of the Singapore Eye Institute, using modifications of the Early Treatment Diabetic Retinopathy Study [32]. All right eyes were free of other major ocular diseases and treatments. Subjects’ demographics including age, mean arterial pressure (MAP) ((SBP + 2DBP)/3), glycated hemoglobin (HbA1C), intraocular pressure (IOP), and axial length (AL) were recorded by trained ophthalmic technicians using standardized protocols [33].

### Vascular oxygen saturation and vessel diameter

**Fig. 1:**
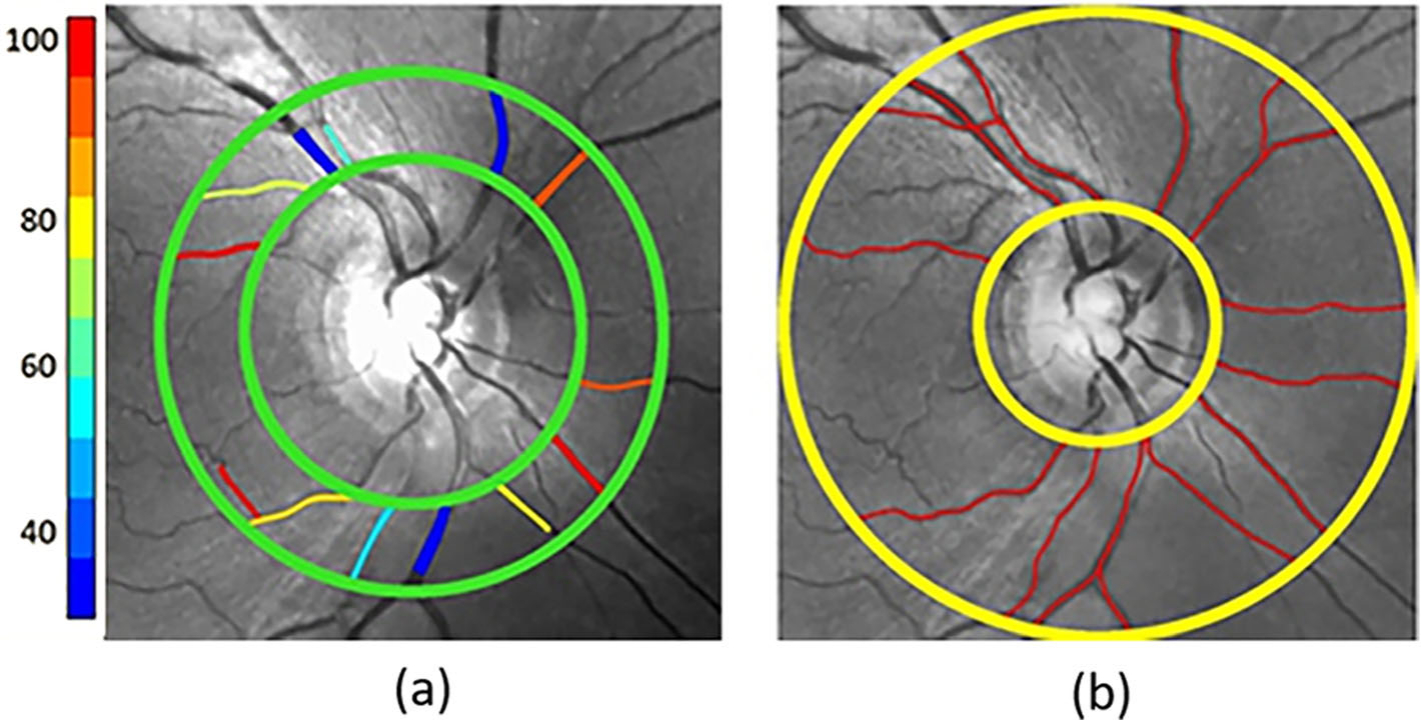
Example of a retinal image at 532 nm in a non-diabetic subject. (**a**) Oxygen saturation measurements in retinal arteries and veins are displayed in pseudo color. Color bar represents hemoglobin oxygen saturation in units of percent. (**b**) Retinal vessel centerlines (red lines) used for tortuosity measurements are overlaid on the retinal image

Retinal vascular oxygen saturation (SO_2_) was measured by our previously developed method [24] using retinal images acquired at two wavelengths (532 nm and 633 nm) by a commercially available scanning laser ophthalmoscope (Optos, Daytona). Our previous study used images from the previous model of the instrument (Optos 200X). From the retinal images, a region of interest was identified within a circumpapillary region centered on the optic nerve head (ONH) and extended between 2 and 3 ONH radii, as indicated by the green circles in Fig. 1a. As previously described [24, 34, 35], vessels were detected using Frangi vesselness filtering [36] and optical density (OD) was determined for each vessel per imaging wavelength as the average ratio of the intensity values inside to outside the vessel. Optical density ratio (ODR) was calculated as OD_633_/ OD_532_. A linear conversion was used to calculate arterial and venous SO_2_ (SO_2A_ and SO_2V_) from the ODR measurements. Diameter (D) of each vessel segment was measured by our previously described method. Distance transformation was used to detect vessel centerlines. Intensity profiles perpendicular to the vessel centerline were obtained at every 7 pixels along the vessels. Full width at half maximum of the intensity profiles were determined from each of the vessel segments and averaged to provide arterial and venous D (D_A_ and D_V_) measurements.

### Vessel tortuosity

Vessel tortuosity was measured based on our previously validated vessel tortuosity index (VTI) [34, 37] within a circumpapillary region that extended between 1.5 and 5 ONH radii, as indicated by yellow circles in Fig. 1b. Retinal vessels were detected using Frangi vesselness filtering to generate a binary image. The endpoints of the vessels were selected on the binary image and distance transformation was used to extract the vessel centerline between the end points. VTI was determined based combination on several tortuosity features as explained previously [37]. Mean and maximum arterial and venous VTI (VTI_A_ and VTI_V_) were determined.

### Data analysis

Statistical analysis was performed with SAS 9.4 (SAS Institute Inc., Cary, North Carolina, USA). Since this was a pilot study in a limited cohort of subjects, sample size was not pre-determined for optimal power. Demographics were compared between groups of subjects using t-test or chi-square test. Linear regression was used to determine the effect of DR stage (ND, NDR, NPDR) on D, SO_2_, VTI after adjusting for covariates including age, MAP, HbA1C, IOP and AL. There was no significant correlation between duration of diabetes and retinal vascular metrics in this sample of diabetic subjects. Statistical tests were 2-sided, and significance was accepted at *P* ≤ 0.05.

## Results

Subjects’ demographics are summarized in Table 1. There were no statistically significant differences in age, MAP, IOP, or axial length among the 3 groups (*P* > 0.1). HbA1C level was higher in the NDR and NPDR as compared to ND (*P* < 0.001). The duration of diabetes was not significantly different between NDR and NPDR subjects (*P* = 0.35).

**Table 1:** Subjects’ demographics. ND, NDR, and NPDR stand for non-diabetic, diabetic without diabetic retinopathy, and moderate/severe non-proliferative diabetic retinopathy, respectively. MAP is mean arterial pressure, HbA1C is glycosylated hemoglobin, and IOP is intraocular pressure

	**ND (*N*** **= 26)**	**NDR (*N*** **= 19)**	**NPDR (*N*** **= 11)**	***P*-value for group difference**
**Age (years)**	57 ± 13	64 ± 12	57 ± 13	0.21
**MAP (mmHg)**	97 ± 14	95 ± 11	95 ± 17	0.87
**HbA1C (%)**	5.6 ± 0.4	7.4 ± 1.4	8.5 ± 2.0	< 0.001
**IOP (mmHg)**	13.9 ± 2.9	16.0 ± 4.1	15.3 ± 2.6	0.11
**Axial Length (mm)**	23.6 ± 0.9	23.5 ± 0.9	23.3 ± 0.3	0.52
**Duration of Diabetes (years)**		7.5 ± 5.4	10.4 ± 9.4	0.35

**Fig. 2:**
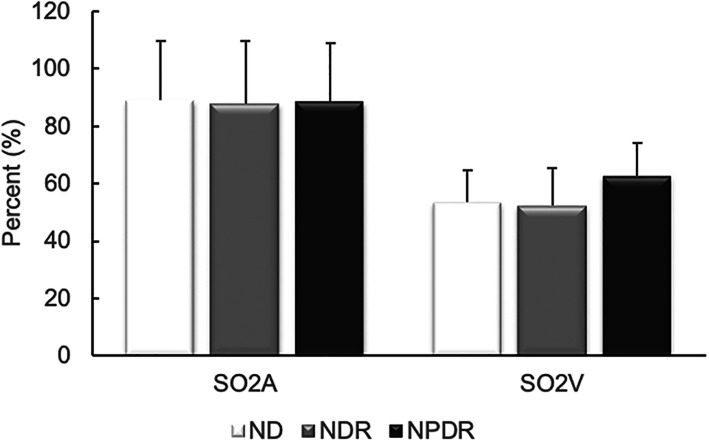
Mean and standard deviation of retinal arterial and venous SO_2_. There was no significant difference in SO_2A_ among ND, NDR, and NPDR groups, while SO_2V_ was higher in NPDR compared to ND and NDR groups

Mean and standard deviation (SD) of retinal arterial and venous SO_2_ are shown in Fig. 2. Mean and SD of SO_2A_ among ND, NDR, and NPDR groups were 89 ± 21%, 88 ± 21% and 89 ± 20%, respectively. SO_2V_ were 54 ± 11%, 53 ± 13% and 63 ± 11% in ND, NDR, and NPDR groups, respectively. There was no significant difference in SO_2A_ among ND, NDR, and NPDR groups (*P* = 0.96), while SO_2V_ was higher in NPDR compared to ND and NDR groups (*P* < 0.05).

**Fig. 3:**
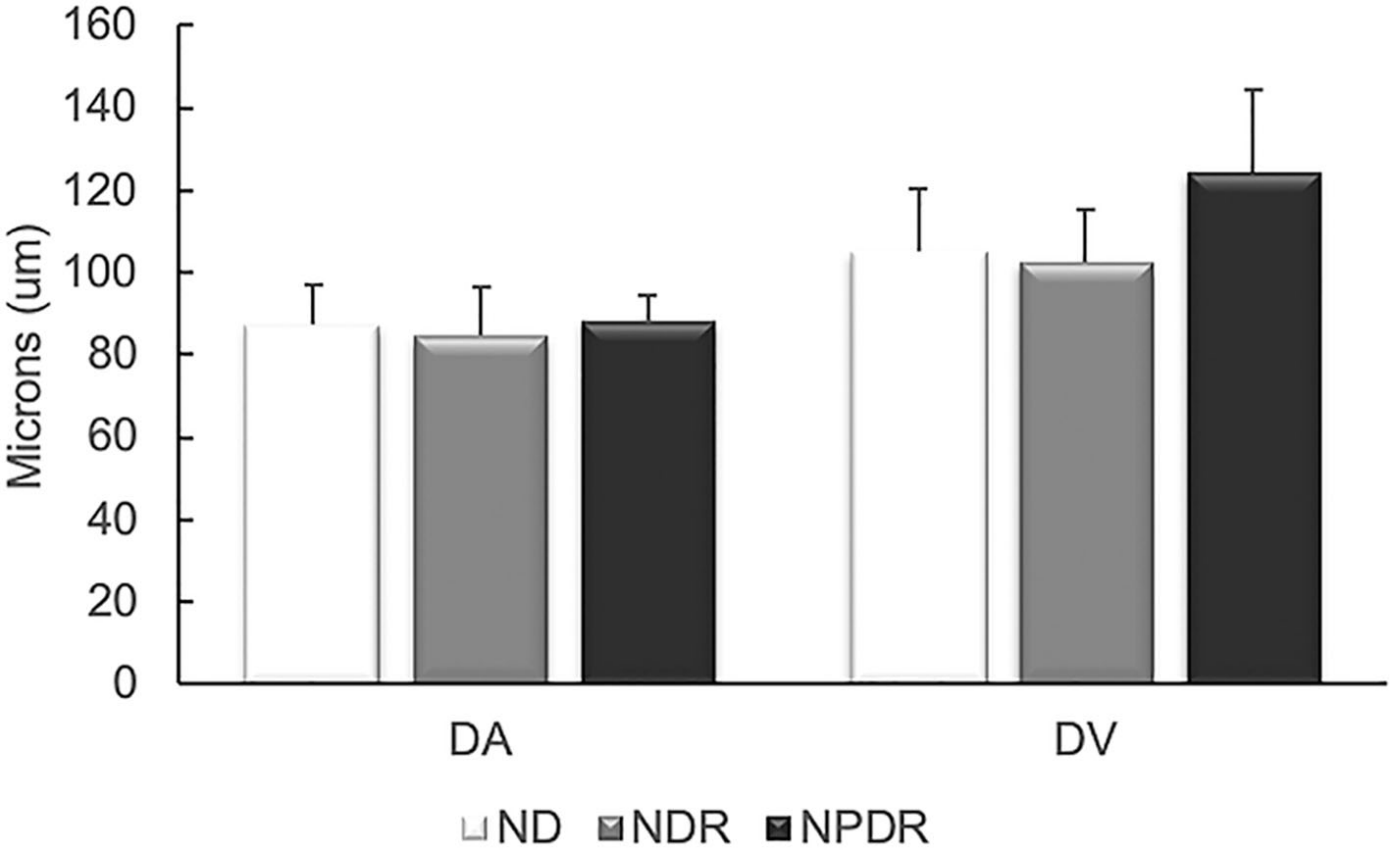
Mean and standard deviation of retinal arterial and venous D. There was no significant difference in D_A_ among ND, NDR, and NPDR groups, while D_V_ was higher in NPDR compared to ND and NDR groups

Mean and SD of retinal arterial and venous D are shown in Fig. 3. D_A_ in ND, NDR, and NPDR groups were 87 ± 10 μm, 85 ± 12 μm and 88 ± 6 μm, respectively. D_V_ were 105 ± 15 μm, 102 ± 13 μm and 125 ± 20 μm in ND, NDR and NPDR groups, respectively. There was no significant difference in D_A_ among ND, NDR, and NPDR groups (*P* = 0.89), while D_V_ was higher in NPDR compared to ND and NDR groups (*P* < 0.05).

**Fig. 4:**
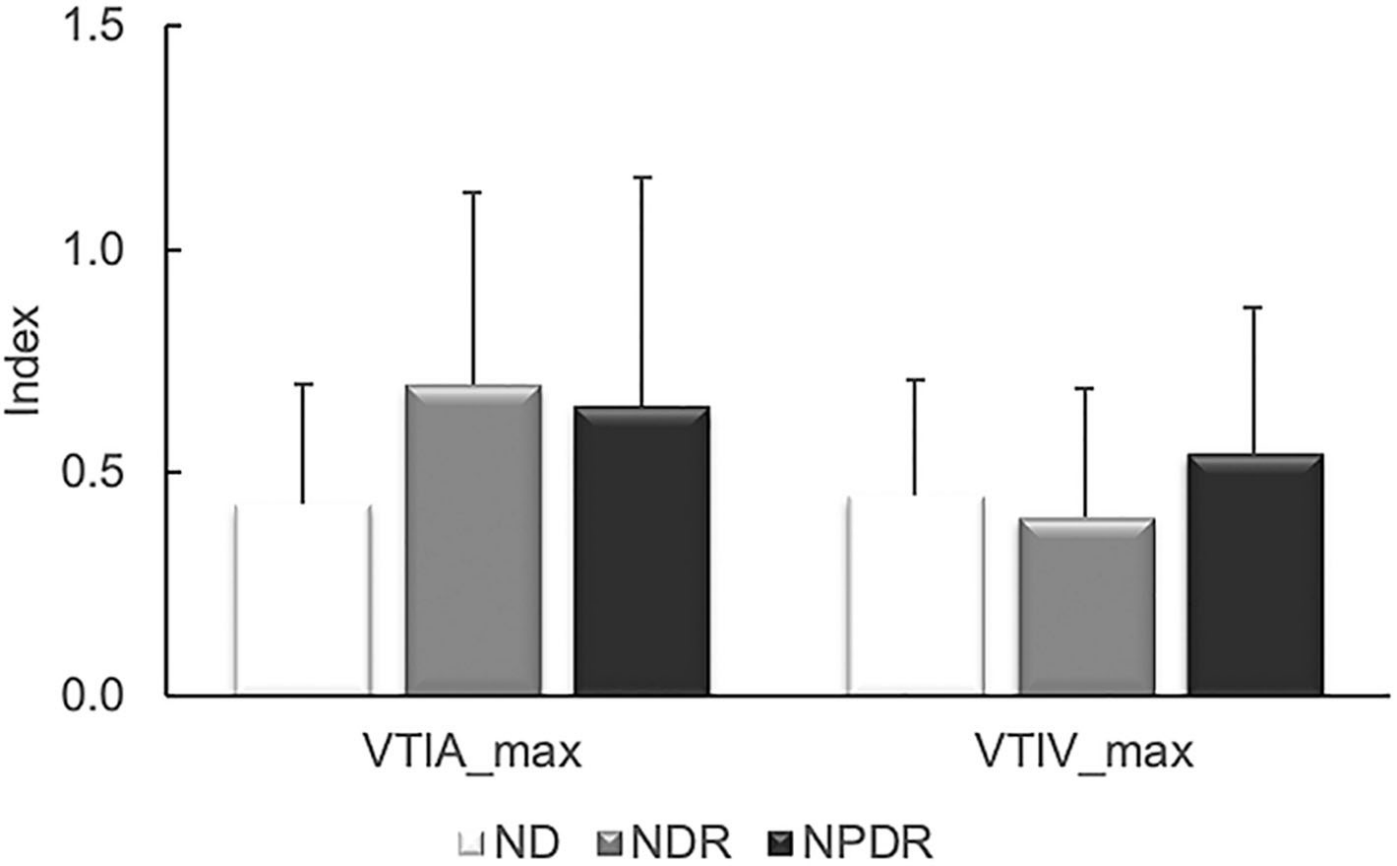
Mean and standard deviation of retinal arterial and venous maximum VTI. Maximum VTI_A_ was higher in NDR and NPDR compared to ND, whereas there was no significant difference in maximum VTI_V_ among groups

Mean and SD of retinal arterial and venous maximum VTI are shown in Fig. 4. Arterial maximum VTI among ND, NDR, and NPDR groups were 0.43 ± 0.26, 0.28 ± 0.19 and 0.65 ± 0.51, respectively. In venules, maximum VTI were 0.44 ± 0.25, 0.40 ± 0.28 and 0.54 ± 0.32 in ND, NDR, and NPDR groups, respectively. Maximum VTI_A_ was higher in NDR and NPDR compared to ND (*P* < 0.03). There was no significant difference in maximum VTI_V_ among groups (*P* = 0.54).

## Discussion

In the current study, we demonstrated increased venous SO_2_, venous diameter, and arterial tortuosity in moderate/severe NPDR in AA subjects. To our knowledge, this is the first report in literature assessing retinal vascular oxygenation and vessel morphology at stages of DR, specifically in AA subjects. The findings have the potential to stimulate future studies aimed at identification of race-specific biomarkers that may help reduce the risk of vision loss due to DR in the AA population.

The findings of increased retinal venous SO_2_ in NPDR, but no significant difference in arterial SO_2_ among DR stages in AA population are consistent with reports of previous studies conducted in primarily white populations [24, 38, 39]. However, other studies found no difference in retinal venous SO_2_ between diabetics with no DR and non-diabetics [31, 40]. Variations in findings are likely due to methodologies, subject demographics, and level of retinopathy.

We found that retinal venous diameter was increased in NPDR, while arterial diameter was not different among DR stages. This finding is in agreement with numerous studies that showed venous vasodilation and variable arterial diameter changes dependent on DR stage [41–47]. This presumable biphasic arterial diameter changes may contribute to the lack of significant findings in arterial diameter in the current study. The finding of increased retinal arterial tortuosity with DR stage is consistent with a previous report [48]. Another study demonstrated that DR subjects with macular edema had elongated and more tortuous retinal vessels than their nondiabetic counterparts [21]. The pathophysiology of why vessels become more tortuous, however, remains unclear. In addition to the theory of increased VEGF secretion due to hypoxia [49], increased vessel tortuosity may occur due to blood flow disturbances leading to loss of vessel endothelium and subsequent failure to maintain the integrity of the vessel wall [50]. Furthermore, this increased fragility of the vessel wall can predispose to hemorrhage which is commonly observed in DR. [51]

The current study is the first report of alterations in retinal vascular SO_2_ in diabetic AA subjects. Although the effect of race on retinal oximetry data has not been previously investigated, a dependence of SO_2A_ on race was demonstrated in a study of both AA and white diabetic subjects [24]. It is not clear whether this finding is due to biological or pigmentation differences between races. However, since the statistical model generated in the previous study adjusted for pigmentation, it is likely that other factors may account for the observed racial differences. Of note, in the current study, the calibration factor used for conversion of light absorption ratios to oxygen saturation was derived based on healthy AA subjects, thus accounted for pigmentation. Comparative studies of retinal vessel diameter and tortuosity between diabetic AA and white subjects are not available. Genetic and racial factors that determine retinal vessel caliber and compliance to adjust to transient blood flow changes may play a role in the susceptibility of subjects for development of DR. For example, a previous genome-wide association study of subjects of European ancestry showed genetic loci associated with retinal vessel diameter [52]. Future studies are needed to identify and compare factors associated with retinal vessel metrics in AA and white populations.

There is limited knowledge of factors that contribute to the observed difference in the prevalence of DR according to race. A large study conducted in the AA diabetic population showed a lack of association between proportion of African ancestry and PDR after adjustment for clinical, demographic, and socioeconomic factors [53]. Furthermore, no genetic loci associated with PDR was identified using admixture mapping in AA subjects. In a case-control study of diabetic AA subjects, duration of diabetes, systolic hypertension and insulin use were found to be risk factors for the development of PDR [54]. Moreover, P-selectin plasma level was associated with DR in the AA population based on both serologic and genetic data [55]. Future studies are needed to investigate association of retinal vascular metrics with stages of DR in the AA population. Such studies have the potential to identify race-specific biomarkers for staging and monitoring progression of DR, thus providing more targeted and effective clinical management of DR in AA subjects.

The current study had limitations. First, measurements were not derived from exactly the same retinal vessel segments and were restricted to a region close to the ONH, thus changes distal to the ONH and in smaller branching vessels were not considered. Second, potential confounders, such as macular edema were not considered which may have contributed to inter-subject measurement variability. Third, imaging was performed with a commercial instrument and the wavelengths were not optimized for oximetry, though previous studies have been performed with a similar instrument [24, 56, 57]. Fourth, data from different racial populations were not available for direct comparison. Fifth, the sample size was small, thus comparisons in which no significant difference was detected may have been due to lack of statistical power (< 15%). Future studies are needed for assessment of retinal vascular oxygenation and vessel morphology in larger and multi-racial cohorts to expand the current findings and potentially identify race-specific biomarkers at progressive stages of DR.

## Conclusions

Overall, the current study showed alterations in retinal vascular oxygenation and morphology in AA subjects at stages of DR. The findings advance our understanding of DR pathophysiology in the AA population and propel future studies to provide race-specific biomarkers for assessment of DR.

## Data Availability

The data generated in the current study are not publicly available due to privacy and security of patients, but can be made available from the corresponding author on reasonable request.

## Abbreviations

DR: Diabetic retinopathy
AA: African-American
SO2: Oxygen saturation
D: Diameter
ND: Non-diabetic
NDR: No clinical DR
NPDR: Moderate/severe non-proliferative DR
VTI: Vessel tortuosity index
MRA: Model reporting area
NHW: Non-hispanic white
VEGF: Vascular endothelial growth factor
AFEDS: African-American eye disease study
HbA1C: Glycated hemoglobin
IOP: Intraocular pressure
MAP: Mean arterial pressure
ONH: Optic nerve head
ODR: Optical density ratio
SD: Standard deviation
